# Three-Dimensional Compound Comparison Methods and Their Application in Drug Discovery

**DOI:** 10.3390/molecules200712841

**Published:** 2015-07-16

**Authors:** Woong-Hee Shin, Xiaolei Zhu, Mark Gregory Bures, Daisuke Kihara

**Affiliations:** 1 Department of Biological Science, Purdue University, West Lafayette, IN 47907, USA; E-Mail: shin183@purdue.edu; 2 School of Life Science, Anhui University, Hefei 230601, China; E-Mail: newxiaoleizhu@gmail.com; 3 Discovery Chemistry Research and Technologies, Eli Lilly and Company, Indianapolis, IN 46285, USA; E-Mail: bures_mark@lilly.com; 4 Department of Computer Science, Purdue University, West Lafayette, IN 47907, USA

**Keywords:** ligand-based virtual screening, three-dimensional similarity, ROCS, USR, 3D Zernike descriptors, Patch-Surfer, PL-PatchSurfer, molecular shape, molecular surface

## Abstract

Virtual screening has been widely used in the drug discovery process. Ligand-based virtual screening (LBVS) methods compare a library of compounds with a known active ligand. Two notable advantages of LBVS methods are that they do not require structural information of a target receptor and that they are faster than structure-based methods. LBVS methods can be classified based on the complexity of ligand structure information utilized: one-dimensional (1D), two-dimensional (2D), and three-dimensional (3D). Unlike 1D and 2D methods, 3D methods can have enhanced performance since they treat the conformational flexibility of compounds. In this paper, a number of 3D methods will be reviewed. In addition, four representative 3D methods were benchmarked to understand their performance in virtual screening. Specifically, we tested overall performance in key aspects including the ability to find dissimilar active compounds, and computational speed.

## 1. Introduction

One of the key steps in the early stage of drug development is finding active compounds, which can serve as molecules that will be further optimized into potential drug candidates. Computational methods that search against a large chemical database, also known as virtual screening [1,2], are commonly used in this stage. Two major categories for computer-based drug discovery are structure-based virtual screening (SBVS) and ligand-based virtual screening (LBVS). SBVS uses the 3D structure of a target receptor when selecting potential active compounds. Molecular docking is a major class of SBVS, which searches the candidate binding pose of a ligand and binding affinity to a pre-defined binding pocket of a target protein [1,3]. Although the number of solved protein 3D structures continues to increase, there are many proteins without known structure that are important drug targets, such as a significant number of G-protein-coupled receptors. In addition, molecular docking methods can take extensive computational time to explore different poses of a compound in a binding pocket of the receptor. Therefore, LBVS methods can be advantageous when the target receptor structure is not available or the chemical database to be screened is very large [2].

LBVS methods perform comparisons between known active compounds of a target and compounds in a database. Several different approaches are used to represent compound structure in LBVS methods:

- 1D representations: e.g., SMILES [4], SMARTS [5]
- 2D representations: e.g., BCI [6], RASCAL [7], MOLPRINT2D [8], and SIMCOMP [9], which use two-dimensional structure fingerprint or graph matching
- 3D representations: e.g., ROCS [10], USR [11], PL-PatchSurfer [12], Blaze [13] that use 3D features such as volume, atomic distances, surfaces, or fields.

Historically, 1D and 2D methods have been used widely in part because chemical molecules are typically represented by 2D molecular formula or 1D fingerprints and because of the relative ease of developing comparison algorithms. However, 1D and 2D methods have a tendency to find mainly close chemical analogues to known active compounds but fail to predict activity differences between them [14]. What is lacking from 1D and 2D methods is obviously 3D structural information of compounds and target proteins. Binding affinity between molecules and target proteins is governed by atomic interactions in the 3D space. Consequently, molecules that have similar 3D shape and properties could share biological activities, even while their 1D and 2D representations are not similar. Therefore, 3D methods have gained more attention recently because of their potential to overcome key limitations of 1D and 2D methods. However, a main challenge of 3D methods is how to treat ligand conformational flexibility. In addition, since multiple ligand conformations are considered, 3D methods require more storage space and computational time compared to 1D and 2D methods [15]. Another important consideration is that a biologically active structure does not always match the lowest energy conformation of a molecule.

In this article, we review 3D-based virtual screening methods. 3D-based representations can be categorized into five classes based on how a 3D structure is represented: atomic distance-, Gaussian function-, surface-, field-, and pharmacophore-based methods. We will discuss each of these five categories in the subsequent section. Finally, we also compare the performance of available representative 3D methods on a benchmark dataset of active and decoy compounds [16].

## 2. 3D Shape-Based Compound Descriptors

In this section, we review 3D compound description and comparison methods. We start with reviewing atomic distance-based methods.

### 2.1. Atomic Distance-Based Methods

One of the simplest ways to describe molecular 3D information is by computing the distribution of atomic distances in compounds. Compared to other classes of 3D methods, atomic distance-based methods are simple and fast since they only calculate the distance between atom pairs for an input coordinate file of a compound.

USR (Ultrafast Shape Recognition) [11] uses statistics of distances between heavy atoms using four key positions of a molecule: the molecule center (ctd), the closest atom from ctd (cst), the farthest atom from ctd (fct), and the farthest atom from fct (ftf). After defining the four points, the method calculates all atom distances from them. Then, it calculates three moments from the four distance distributions: (1) the mean distance; (2) the variance of the distribution; and (3) the skewness of distribution. Therefore, a molecule has 12 values (four distribution times three moments) to describe a 3D shape. The similarity of two molecules, A and B, is given as an inverse of the Manhattan distance of the 12 values (Equation (1)):

(1) $$S_{\text{AB}}=\frac{1}{1+\frac{1}{12}\sum_{i=1}^{12}\left|A_{i}-B_{i}\right|}$$

Another method, ESHAPE3D [17], also uses pair-wise heavy atom distances. These distances are stored in a single matrix, where rows and columns denote each atom. Then, the molecular shape is characterized by eigenvalues computed after diagonalizing the matrix. The similarity score between two compounds, each of which is represented by a fingerprint that uses eigenvalues, is calculated as the inverse of the distance between their fingerprints.

The disadvantage of distance-based methods is that it can be difficult to encode physicochemical features of a molecule. All atom pairs are treated in the same way when comparing molecules. Therefore, this methodology cannot discriminate between structures with a similar shape but with differences in functional groups [18]. To resolve this problem, atom types or pharamacophoric constraints are introduced in USR variants. USRCAT (Ultrafast Shape Recognition with CREDO Atom Types) detects five pharmacophore features, hydrophobic, aromatic, hydrogen bond donor, and hydrogen bond acceptor, and calculates 12 components for all atoms and for every feature [18]. Therefore, one molecule contains 60 values (five times 12 components). Intrinsic limitations of distance-based methods are that 3D information of molecules is not fully captured, for example, special positions of atoms and relative positions among them are not described and enantiomers cannot be distinguished.

### 2.2. Gaussian Function-Based Molecular Shape Description Methods

This class of methods computes the similarity of molecules as volume overlap of molecules after superimposition. The Gaussian function (Equation (2)) is widely used to represent molecular volume and shape. The advantages of Gaussian function are that its derivative and integral can be analytically computed and that the product of two Gaussian functions becomes also a Gaussian function. The atomic density of a molecule is given as a spherical Gaussian function [19]:

(2) $$\rho_{i}(r)=p_{i}\text{exp}\left[-\pi {\left(\frac{3p_{i}}{4\pi \sigma_{i}^{3}}\right)}^{\frac{2}{3}}{\left(r-R_{i}\right)}^{2}\right]$$

where **r** and **R**_i_ are a coordinate of a surface point and the atomic coordinate i in the Euclidean space, respectively. σ_i_ is a van der Waals radius of the atom i and p_i_ is the scaling factor of the Gaussian function. In most applications of the Gaussian function to calculate molecular volume, *p_i_* is set to 2√2. The volume of an atom *i* can be calculated as an integral of ρ_*i*_ for over the space.

The volume overlap between two molecules after superposition, A and B, is calculated with Equation (3). The summation is computed over all the atoms in each molecule.

(3) $$V_{\text{AB}}=\sum_{i\in A}\sum_{j\in B}\int \rho_{i}(r)\rho_{j}(r)dr=\sum_{i\in A}\sum_{j\in B}p_{i}p_{j}\text{exp}\left(-\frac{\sigma_{i}\sigma_{j}}{\sigma_{i}+\sigma_{j}}\right){\left(\frac{\pi }{\sigma_{i}+\sigma_{j}}\right)}^{\frac{3}{2}}$$

Two molecules need to be superimposed before the volume overlap is computed. This is a disadvantage of this type of methods since superimposition of molecule takes time and also the similarity score severely depends on superimposition, which is not always trivial particularly for molecules of spherical shape.

The most widely used program that uses Gaussian function is ROCS (Rapid Overlay of Chemical Structures) developed by OpenEye [10]. ROCS tries to find a superimposition of a query to a template molecule that maximizes volume overlap between them. Then, it defines a similarity between the two molecules as a volume Tanimoto coefficient:

(4) $${\text{Tanimoto}}_{\text{query},\text{template}}=\frac{V_{\text{query},\text{template}}}{V_{\text{query}}+V_{\text{template}}-V_{\text{query},\text{template}}}$$

where V_query_, V_template_, and V_query,template_ are the volumes of query molecule, template molecule, and the overlapped region after superimposition, respectively. If the molecules share more similar structure, higher Tanimoto coefficients can be obtained.

In addition to shape similarity, ROCS can calculate chemical similarity between molecules after superimposition. The chemical type of an atom is assigned by Implicit/Explicit Mills Dean color force field [20]. The atoms types considered are hydrogen bond donor, hydrogen bond acceptor, anion, cation, hydrophobic, and rings. The chemical similarity between two molecules is also quantified with a Tanimoto coefficient.

Since ROCS only uses shape to overlay molecules, this can lead to issues when the ligand database contains many similar molecules [11]. Moreover, the alignment algorithm ROCS uses, a SIMPLEX algorithm, cannot guarantee finding the optimal overlap. Therefore, ROCS does not guarantee the best superposition between two molecules. To accommodate this problem, physicochemical properties such as electrostatic field are introduced in other programs in the superimposition stage of molecules.

MolShaCS [21] introduced an empirical charge distribution function. The base function is also Gaussian, but the van der Waals radius, σ_*i*_ in Equation (2), is replaced to the atomic charge. The overlap between two molecules, A and B is calculated as Equation (5).

(5) $$V_{\text{AB}}=w_{1}\sum_{i}\sum_{j}\int \rho_{i}(r)\rho_{j}(r)dr+w_{2}\sum_{i}\sum_{j}\int \phi_{i}^{\text{pos}}(r)\phi_{j}^{\text{pos}}(r)dr+w_{2}\sum_{i}\sum_{j}\int \phi_{i}^{\text{neg}}(r)\phi_{j}^{\text{neg}}(r)dr$$

MolShaCS separates the charge distribution function into positive (φ^pos^) and the negative part (φ^neg^). The similarity between two molecules is calculated as the Hogkin’s index:

(6) $${\text{Hogkin}}_{\text{query},\text{template}}=\frac{{2V}_{\text{query},\text{template}}}{V_{\text{query}}+V_{\text{template}}}$$

The two methods introduced so far superpose two molecules to maximizing volume (and electrostatic potential for MolShaCS) overlap between them. Alternatively, there are methods that use a transformation matrix which is obtained from aligning molecular feature frames. ShaEP (reminiscent of Shape and Electrostatic Potential) [22] represents a molecule with a so-called molecular 3D field-graph. In the graph, vertices are defined for heavy atoms of a molecule following directions of hybridized orbitals in atoms that are not used for forming a covalent bond. These vertices are located at a distance of σ + h Å from the origin atom where σ is van der Waals radius of the atom and h is an adjustable parameter. Additional vertices considered are normal to planar rings that have more than four atoms. Every vertex has charge and shape density information. The charge density of a vertex is calculated as

(7) $$\phi_{E}=\frac{1}{4\pi \varepsilon_{0}\varepsilon_{r}}\sum_{i}\frac{q_{i}}{d_{i}}$$

where q_i_ is a charge of an atom, *d_i_* is a distance from the atom and the vertex, ε_r_ is the relative permittivity, which can be given by a user, and (4πε_0_)^−1^ is the Coulomb constant. The shape density of vertex is a summation over all atoms of the Gaussian density function, (Equation (2)). The graph for a molecule is constructed by fully connecting vertices.

A transformation matrix is obtained for matching graphs of two molecules. For matching nodes, electrostatic potential difference should not be larger than a user-defined threshold and the dot product of the shape density vertex should be no less than a threshold given by the user. In addition, compatible edges should have similar length with a length difference less than 1 Angstrom. After obtaining candidate transformation matrices, the similarity scores are given as a sum of Hogkin’s similarity index (Equation (6)) of shape and the electrostatic potential. Shape overlap is calculated following Equation (3). Overlap of electrostatic potential is obtained as

(8) $$V_{\text{exp},\text{AB}}=\sum_{k\in A}\text{exp}[-\beta \left\|\phi_{E,k}-\phi_{E}^{B}(r_{k})\right\|]+\sum_{l\in B}\text{exp}\left[-\beta \left\|\phi_{E,l}-\phi_{E}^{A}(r_{l})\right\|\right]$$

where *k*, *l* are vertex of molecule A and B, *r_k_* and *r_l_* are the coordinate of them. β is set to one by default. After scoring all transformation matrices, the highest score will be assigned to the query molecule pair.

SHAFTS (SHApe-FeaTure Similarity) [23] is another method that uses a transformation matrix after aligning feature frameworks of two molecules. First, it detects pharmacophores of a given molecule conformation. The pharmacophores are hydrophobic centers, positive charge centers, negative charge centers, hydrogen bond acceptors and donors, and aromatic rings. Then, the program connects all combinations of three points to make triangles and store geometric information of them in a hashing table. The transformation matrix is constructed by least square fitting of triangles from query and template molecules. After overlaying the molecules, volume overlap is calculated following Equation (3). Feature overlap is obtained as below.

(9) $$F_{\text{AB}}=\sum_{f}\sum_{i\in A}\sum_{j\in B}\text{exp}\left[-2.5{\left(\frac{d_{\text{ij}}}{R_{f}}\right)}^{2}\right]$$

**Figure 1:**
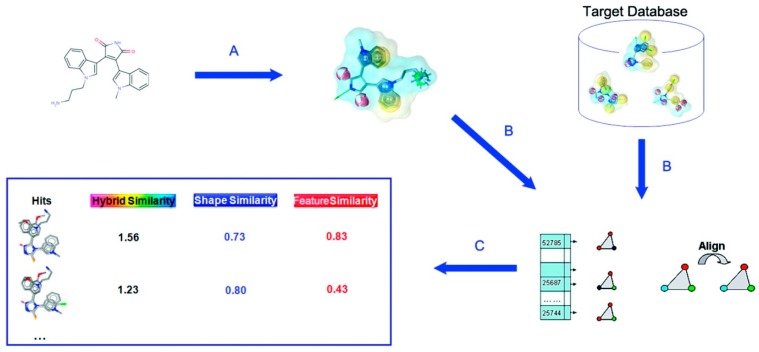
Schematic illustration of SHAFTS procedure. (**A**) Generate pharmacophore feature points of selected active molecule; (**B**) Search a database by superimposing feature triplet; (**C**) Rank compounds by the similarity score. Reprinted with permission from [23]. Copyright (2015) American Chemical Society.

*f* is a pharmacophore feature, *i* and *j* are feature points. *d_ij_* is a distance between feature points *i* and *j* after superimposition, and *R_f_* is a tolerance. The similarity of two molecules is given as Equation (10). Procedure of SHAFTS is illustrated in Figure 1.

(10) $$S_{\text{AB}}=\frac{V_{\text{AB}}}{\sqrt{V_{A}V_{B}}}+\frac{F_{\text{AB}}}{\sqrt{F_{A}F_{B}}}$$

### 2.3. Surface-Based Molecular Shape Description

An alternative for molecular shape description is to consider the surface of molecules. Using surface representation, ligand binding can be considered as a matching complementary surface of protein binding site and a ligand surface [24]. Since 3D structures of proteins and ligands are primarily described by coordinates of atom positions, a surface needs to be constructed from coordinate files. Surface-based LBVS methods are characterized by two factors, how a surface is constructed and the features used to describe molecular surface.

MSMS [25] is one of the most popular programs for constructing molecular surface. MSMS generates molecular surface by triangularization. Triangular representation is composed of a set of vertices and a group of triangular patches that connect vertices. MOLPRINT3D [26] is an example of programs that uses MSMS for computing surface of compounds. Points on compound surface are characterized by interaction energies using GRID [27], *i.e.*, Lennard-Jones, hydrogen bonds, and electrostatic potential, against probe atoms such as methyl CH_3_ carbon, sp^3^ NH cation, amide NH_2_ group, carbonyl oxygen atom, and anionic phenolate oxygen atom, respectively. These energy values on the surface are binned and then each surface point is assigned with a vector that consists of energies of the position and its neighboring points. Similarity of two molecules is quantified by comparing the vectors of surface points.

Similar to MOLPRINT3D, LASSO [28] characterizes a molecular surface with the interacting surface point type (ISPT). ISPT is composed of 23 chemical types, such as hydrogen bond donor, lone pair electron, π electron on sp^2^ carbon, and halogen. On the surface of a molecule, surface points are generated following the definition of each ISTP. By counting numbers of each ISPT on the surface, a molecule has a feature vector with 23 components. Each number at each component represents how many interaction points of the type are generated on the ligand surface. A similarity between two ligands is predicted by neural network, which is trained by a subset of the DUD compound dataset [29].

An alternative surface representation is computed from a Voronoi diagram [30]. A Voronoi diagram partitions a given space with points so that a partition between a pair of points is placed at the same distance from them. Wilson and coworkers presented a molecule comparison method using α-shape [31]. The computational step of α-shape uses a probe sphere, also called an eraser, of a certain radius. Two atoms are connected by an edge if the eraser probe cannot go through the atom pair indicating that they are close to each other. This concept is expanded to a triplet of atoms; if the eraser can contact all the atoms at the same time, a triangular face is created with three vertices connecting the three atoms. Computation of α-shape is faster than conventional surface generation methods that use space-filling with spheres centered at atomic coordinates. After the surface of a molecule is obtained, similarity of two molecular surfaces can be quantified by computing the similarity of two distribution of distances and angles of normal vectors of all the pairs of facet centers [32].

BetaDock [33] uses another surface representation called the β-shape that is generated also from a Voronoi diagram. β-shape uses similar procedure as α-shape but their differences include that β-shape is able to robustly construct surface for a set of spheres of different radii, e.g., a compound with different heavy atoms, which α-shape cannot handle properly [34]. The BetaDock program represents pocket with β-shape and docks a ligand at the surface of the pocket. Thus, BetaDock is a SBVS method but mentioned here because it uses a different surface representation which can be readily applicable for LBVS methods.

Lastly, we introduce moment-based molecule surface representation. Moment-based methods use a mathematical series expansion and allow compact representation of molecular surface because essentially a surface shape can be specified by coefficient values of the expansion. PARAFIT [14] describes molecular surface with spherical harmonics. Setting the center of mass of a molecule at the origin of the coordinate system, the surface of the molecule is characterized as radial expansion:

(11) $$r(\theta ,\varphi )=\sum_{l=0}^{L}\sum_{m=-l}^{l}a_{\text{lm}}y_{\text{lm}}\left(\theta ,\varphi \right)$$

where (θ, φ) are spherical coordinates and y_lm_ is a spherical harmonic function. a_lm_ and L are an expansion coefficient and order of expansion, respectively. The a_lm_s, are considered as molecular fingerprints to describe molecular shape. The distance between two molecules, A and B, is calculated as below.

(12) $$D_{\text{AB}}=A_{L}^{2}+B_{L}^{2}-2A_{L}B_{L}$$

where $A_{L}=\sum_{l=0}^{L}{\left[A_{l}^{2}\right]}^{\frac{1}{2}}$, and $A_{l}=\sum_{l=0}^{L}{\left[a_{\text{lm}}^{2}\right]}^{\frac{1}{2}}$. Because a_lm_ are expansion coefficients for spherical harmonics, the distance D in Equation (12) are rotationally invariant.

Our group proposed 3D Zernike moment-based molecular surface description method [35,36]. The 3D Zernike function is defined as follows:

(13) $$Z_{\text{nl}}^{m}(r,\theta ,\varphi )=R_{\text{nl}}(r)Y_{l}^{m}(\theta ,\varphi )$$

where *Y_m_^l^* is the spherical harmonics and *R_nl_*(*r*) is the radial function. *m* and *l* are integers that have ranges −1 < m < 1 and 0 ≤ 1 ≤ n. After generating Connolly surface of a molecule [37], the surface is mapped on the 3D grid and voxelized, which is considered as the 3D function *f*(*x*) to be expanded. Then, 3D Zernike moments of surface shape, *f*(*x*), are computed as Equation (14).

(14) $$\Omega_{\text{nl}}^{m}=\frac{3}{4\pi }\int f(x)\bar{Z_{\text{nl}}^{m}(x)}dx$$

To obtain rotational-invariant descriptors, a norm, $F_{\text{nl}}=\left\|\Omega_{\text{nl}}^{m}\right\|$ is computed. This vector, *F_nl_* is called 3D Zernike Descriptor (3DZD). 3DZD is mathematically superior to spherical harmonics because it has the radial function, which can incorporate distance information of each surface point from the molecular center. Thus, complicated, non-star-like structures can be also properly represented. To compare the similarity between molecules, correlation coefficients or Euclidean distance of two *F_nl_* is calculated.

3DZD can be directly applied to represent overall (global) shape and physicochemical properties of a molecule [38]. 3DZD can be also used to describe local properties of a molecule by first segmenting surface into patches as introduced in Patch-Surfer [39] and Patch-Surfer2.0 [40]. Unlike methods that describe global properties of a molecule as a whole, which we call later global property-based methods or global methods, Patch-Surfer and Patch-Surfer2.0 try to find similar localized regions between molecules. Thus, later we called Patch-Surfer a local property-based method or local method because it explicitly compares local regions of molecules. The motivation of using patches is to be able to recognize local similarity of molecules, including molecules in different conformations. Segmented surface patches are described with several features, including surface shape, hydrophobicity, the electrostatic potential, and visibility (concavity). All these features can be expressed by 3DZD. To represent a physicochemical property of a molecule, its values are mapped on the surface, which are considered as the 3D function and 3DZD are computed in the same way as Equation (14). In Patch-Surfer2.0, a new feature that describes relative position of each patch in the surface was added. The feature similarity between patches is calculated as the Euclidean distance of 3DZDs. Then, the overall similarity between patches from two molecules is the weighted sum of all feature similarities. Finally, based on the similarity of each patch pair, similarity of two molecules is computed by optimizing corresponding patches in the two molecules so that similarity is maximized (or the distance is minimized) [41].

**Figure 2:**
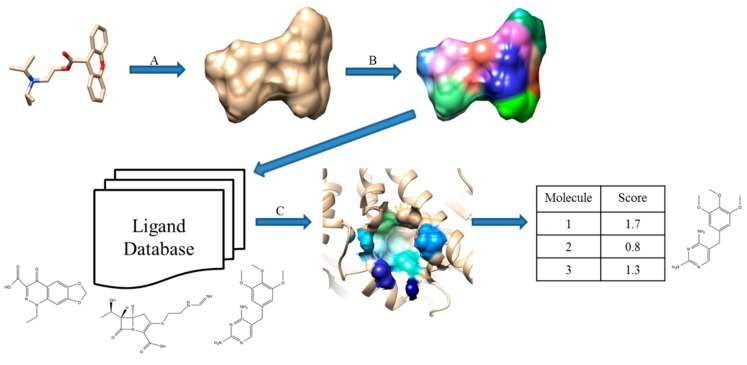
Schematic illustration of PL-PatchSurfer. (**A**) Generated molecular surface of a ligand; (**B**) Patch generation and 3DZD of physicochemical feature calculation; (**C**) Searching binding ligands from a ligand database by finding complementary patch pairs with the query receptor pocket.

Patch-Surfer and Patch-Surfer2.0 were originally developed for comparing query pockets in protein surface against known binding pockets in a database to predict ligands for the query pocket. This approach was further extended in PL-PatchSurfer [12], which compares a query protein pocket against known ligands. The pocket and ligand surfaces are segmented into patches and matched in the same way as performed in Patch-Surfer and Patch-Surfer2.0 except that patches with complementary electrostatic potential are matched and additional features, hydrogen-bonding acceptors and donors, are also considered. Schematic illustration of PL-PatchSurfer is shown in Figure 2.

To summarize, the main advantage of surface-based methods is that molecular global and local similarity can be identified that are independent from atomic details of molecules. Also, representing physicochemical properties on molecular surface is intuitive and technically easy to implement.

### 2.4. Field-Based Methods

The fourth category we introduce is field-based methods. As we have seen so far, molecular properties such as electrostatic potential are assigned to atoms or surface points in volume-based and surface-based methods. In contrast, field-based methods compare the molecular field itself.

**Figure 3:**
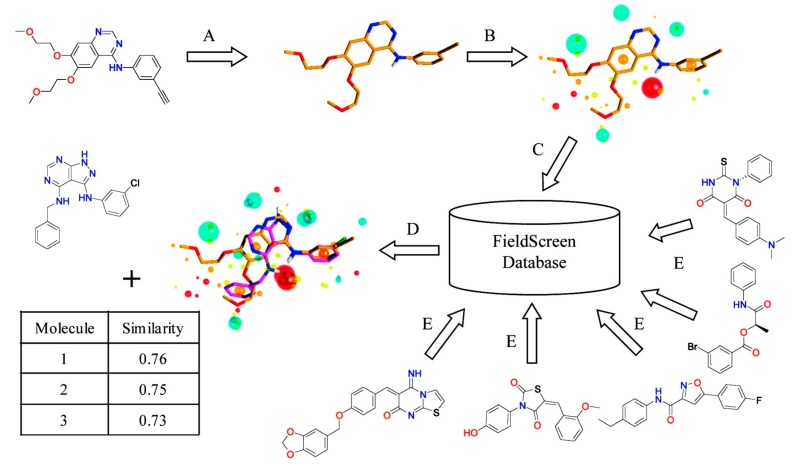
Schematic view of Blaze, which was previously called Fieldscreen. (**A**) A known active molecule is selected as a query; (**B**) Field points generation; (**C**) Searching a ligand database; (**D**) Rank compounds in the database by the similarity score. Reprinted with permission from [13]. Copyright (2015) American Chemical Society.

Cheeseright *et al.* developed an algorithm called Blaze [13,42]. They calculate four molecular fields for the 3D space around a target molecule by rolling a probe sphere on the 3D grid. In addition, van der Waals potential described by the Morse potential, the positive and the negative part of the electrostatic potential, and hydrophobicity are considered. To simplify the field representation, grid points that show similar energy values are merged and they are represented by the maximum points of each field. The similarity between molecules is calculated by the number and the size of the field points matched after aligning molecules so that the overlap between grid points is maximized. Figure 3 illustrates the Blaze workflow.

BRUTUS [43] focuses on the electrostatic potential to represent the field around molecules. Because selection of partial charges is important for aligning molecules, it uses four types of empirical charges: Gasteiger-Huckel, Gasteiger-Marsili, MMFF94, and MOPAC6.0 MNDO/ESP. The electrostatic potential of a ligand is calculated by CoMFA [44] with a 1.0 Å grid. The similarity between ligands is calculated by the Hogkin index using electrostatic potentials inside the van der Waals surface.

Field-based methods compare how the presence of molecules affects to other molecules in the space but do not directly compare 3D structure of molecules. Parretti *et al.* reported that the alignment using electrostatic potential is often different from the optimal superimposition [45]. However, the flip side of the coin is that because the alignment is not based on actual shape, it has the ability to find new leads that are structurally different from a template ligand.

### 2.5. Pharmacophore-Based Methods

Pharmacophore is an abstract depiction of molecular features that is essential for protein-ligand binding [46]. This category generally starts with identifying pharmacophore points that include features such as hydrogen bonding, hydrophobicity core, cation, and anion. After finding the points, three or four of the points are connected to form a triangle or tetrahedron based on the description method (3-point or 4-point pharmacophore keys). These representations are further encoded into strings that have information of the selected features and the distances of the figure edges. Similarity calculation between database and a query molecule is given by Tanimoto Coefficient or other metrics.

FLAP [47] searches pharmacophoric features by calculating energy values by GRID [27]. From discrete points calculated by GRID, all combinations of 4-point pharmacophores (quadruplets) are produced. During the search process, all of the quadruplets from a query ligand are compared with all quadruplets of database. The quadruplets are assigned as matched if the distance differences between the edges of tetrahedrons are within tolerance. Matched quadruplets are then used for aligning molecules. Tanimoto coefficient between molecular fields of aligned molecules is used as a score.

Tuplets [48,49] from Tripos, encodes 2-, 3-, and 4-point pharmacophores. The distance of edge is binned with 1 Å bins, and pharmacophore mutiplets are stored into bit strings. The similarity between a query and database is calculated by Tanimoto coefficient or cosine similarity.

Although pharmacophore-based methods have been widely used, it was discussed that there are a couple of limitations to this approach, which include development of good scoring metrics and that there is no clear way to generate pharmacophore keys [50]. It is known that similar pharmacophores can lead to identifying very different molecules [51,52].

## 3. Benchmark Study

Here, we report benchmark results of four representative LBVS methods among those introduced above.

### 3.1. Benchmark Set

We used a benchmark dataset of active and decoy compounds collected by Cleves and Jain [16]. They identified 979 human drugs from the National Drug Code Directory. After assigning the drugs to their targets, they selected 48 receptors that have the largest number of competitive small molecule drugs. The 48 targets are then further clustered into 22 targets by all-by-all ligand similarity calculation. These 22 targets are important therapeutic targets, such as viral pathogens, steroid receptor targets, and GPCRs. All targets are associated with four to 20 drug molecules. The dissociation constants of the drugs vary depending on targets. For example, K_d_ of clotrimazole-lanosterol demethylase complex is 0.1 μM, and that of fentanyl-muscarinic receptor is 12 nM. Jain selected two or three diverse compounds as templates from active compounds for LBVS for each target. The same decoy set was used for all the target proteins. Compounds for the decoy set were gathered by Rognan from the Advanced Chemical Directory database with criteria that they have drug-like properties (the number of hydrogen bond donors is 0–2; the number of hydrogen bond acceptors is 2–9; the number of rotatable bonds is 2–8, the number of rings is 1–4; and the molecular weight is in the range of 250–500) [53]. The number of decoy molecules is 850. Among 850 ligands in the Rognan decoys, we excluded eight molecules because the OMEGA program [54] failed to generate multiple 3D conformations (see next Section 3.2.).

The performance of four programs listed below was compared in their ability to identify active compounds by comparing template active compounds against a mixture of other actives and the 842 common decoys. Since each target has two to three active compounds, the comparison was performed from each active compound and the average accuracy value was reported. Compared compounds were ranked by its similarity to the template active compounds and the enrichment factors (EF) and area under receiver operating characteristic curve (AUC) values were reported as evaluation metrics.

EF of top α% subset is calculated as Equation (15):

(15) $${EF}_{\alpha \%}=\frac{N_{Actives,\alpha \%}/N_{Databsse,\alpha \%}}{N_{Actives}/N_{Database}}$$

where *N_Actives_* is the number of actives in the whole database, and *N_Database_* represents the total number of molecules in the database. The subscripts α% means that only top α% of molecules selected by a program are considered. EF evaluates concentration of active molecules at earlier ranks by a virtual screening method. The highest EF will be obtained if all actives are selected at the top ranks. In this benchmark, we examined EFs at 2%, 5%, and 10%. A smaller cutoff (e.g., 2%) is used to evaluate early enrichment of actives, which is practically more meaningful than a larger cutoff particularly when a database is very large. As such, 5% and 10% are used as an evaluation metric of virtual screening [55,56,57] and sometimes also for real virtual screening experiments [58,59,60].

Receiver operating characteristic (ROC) curves illustrate the performance of virtual screening programs by considering ratio of found actives and decoys at each ranks of a search. The performance of a method is quantified by computing the area under the ROC curve (AUC). The AUC ranges from 1.0 for the perfect search where all actives are found before any decoy compounds to 0.0 for the opposite case. Although AUC is often used as a virtual screening evaluation metric, it should be noted that AUC considers the entire database ranking and does not sufficiently reward methods that can recognize actives in early ranks [61].

### 3.2. OMEGA

To consider ligand flexibility (*i.e.*, alternative conformations of ligands), we used OMEGA [54] to generate multiple conformations for all compounds in the dataset. The “ewindow” parameter was set to 15 kcal/mol, and RMSD between compounds was set to 0.5 Å if the number of ligand torsion is between zero and four, 0.8 Å if it was between five and nine, and 1.0 Å if it was higher than nine. To check the performance with different number of ligand conformations, “maxconfs” was set to 50, 10, or 5.

### 3.3. Programs Benchmarked

Four methods were compared: USR [11] from the atomic distance-based methods, ROCS [10] from the Gaussian function-based methods, and global 3D Zernike descriptor (GZD) [38], and Patch-Surfer [12] from the surface-based methods. The first three methods describe molecules with their global features (global property-based methods) whereas Patch-Surfer finds similarity in local regions of molecules (local property-based methods).

To compute GZD for a compound, first, a surface was constructed by MSMS [25]. Then, GZD was calculated through Equations (13) and (14), which resulted in vectors of 121 values. With GZD, only the surface shape was represented. The similarity of two conformations was quantified by correlation coefficients between their 3DZDs. For Patch-Surfer, 3DZD of shape, electrostatic potential, hydrophobicity, and hydrogen bonding features were calculated for a molecule. Because Patch-Surfer was not originally designed to calculate similarity between ligands (rather for binding pockets), for this benchmark the scoring function was modified to include the difference of the number of patches on the molecular surface, which takes size differences of the molecules into account. Details of the scoring terms are explained in [12,40].

## 4. Results and Discussion

### 4.1. Overall Results

The performance of the four programs was compared in terms of enrichment factor (EF) values and area under the curve (AUC) (Table 1). We also varied the number of conformations generated for each compound to examine the effect on performance. Intuitively, generating an increased number of conformations is better for capturing molecular flexibility [62] but this comes with an increased cost in computational time.

**Table 1:** Enrichment factors and area under the curve (AUC) values of the methods with different maximum numbers of conformations generated.

	EF_2%_	EF_5%_	EF_10%_	AUC
**50 Conformations**				
USR	10.0	6.2	4.1	0.76
GZD	13.4	8.0	5.3	0.81
PS	10.7	6.6	4.9	0.78
ROCS	20.1	10.7	6.2	0.83
**10 Conformations**				
USR	9.6	6.3	4.1	0.75
GZD	13.5	7.9	5.0	0.78
PS	10.6	6.5	4.9	0.78
ROCS	18.8	9.7	6.0	0.81
**5 Conformations**				
USR	9.6	6.1	4.1	0.75
GZD	12.9	7.3	4.9	0.75
PS	10.3	6.5	4.8	0.77
ROCS	18.2	9.4	5.9	0.80
**1 Conformation**				
USR	8.8	5.8	4.0	0.70
GZD	12.1	7.4	4.9	0.75
PS	10.3	6.4	4.7	0.77
ROCS	15.9	8.5	5.6	0.79

Using 50 conformations (Table 1), ROCS showed the best performance in both EF and AUC values. The ranking of methods in all the metrics is the same, ROCS was the best and GZD, PS, and USR followed in this order. The ROCS method showed an EF_2%_ value that is about twice as high as USR and PS, and about one-and-a-half times higher than GZD. As the percentage of EF increased, the gap between ROCS and other methods decreases, however, their rank order did not change. AUC values of ROCS, GZD, PS, and USR were 0.83, 0.81, 0.78, and 0.76, respectively.

To test the statistical significance, we employed pairwise Student’s *t*-test to the EF_2%_ results with 50 conformations in Table 1. The *t*-values are shown in Table 2. ROCS performed significantly better than all the methods studied (Student’s *t*-test *p*-value < 0.05) while the other three methods cannot be distinguished. If we extend the *p*-value threshold to < 0.1, then GZD’s performance is significantly better than USR.

**Table 2:** Pairwise student *t*-test between pairs of the methods.

	ROCS	GZD	PS
GZD	**2.319**	-	-
PS	**3.544**	1.118	-
USR	**3.750**	1.403	0.360

The *t*-values of performance of individual methods are shown. *t*-values at *p*-value = 0.1 and *p*-value = 0.05 are 1.302 and 1.682, respectively. A *t*-value are shown in bold if it is larger than 1.682 (*i.e.*, statistically significant at *p*-value = 0.05) and underlined if it is larger than 1.302 (*i.e.*, statistically significant at *p*-value = 0.1).

As the number of conformations is decreased, both EF and AUC values were reduced, as expected. Although ROCS performed best in all EF cutoffs and AUC, its performance dropped much faster than the other three methods as the number of conformations decreased. Comparing the 50 and the one conformation cases at EF_2%_, ROCS changed from 20.1 to 15.9 (20.9% decrease), USR changed from 10.0 to 8.8 (12.0% decrease), GZD changed from13.4 to 12.1 (9.7% decrease), and PS changed from 10.7 to 10.3 (3.7% decrease).

Among the four methods compared, PS is the only method that compares local shape of molecules. This difference is exhibited in the results of this experiment in the smallest decrease of the performance (3.7%) by PS as the number of conformations is reduced. USR showed similar performance when 50 to five conformations were generated but it showed a substantial drop when only one conformation was used. An extreme example is opioid receptor μ. With 50 conformations, the three global shape comparison methods, ROCS, GZD, and USR showed EF_2%_ of 19.93, 9.96, and 4.98, respectively, where PS’s EF_2%_ was 9.96, the same value as GZD. However, when the maximum number of conformations was reduced to 10, EF_2%_ of all ROCS, GZD, and USR dropped to zero while that of PS still remained at 9.96.

These results are consistent with previous comparative studies on the DUD dataset [63]. According to the study [63], ROCS performs best among molecular 3D shape comparison programs including USR. The paper also reported that the differences in relative enrichment factors between ROCS and other programs decreases as larger cutoffs were used for the enrichment factor as our benchmark results in Table 1 show.

**Figure 4:**
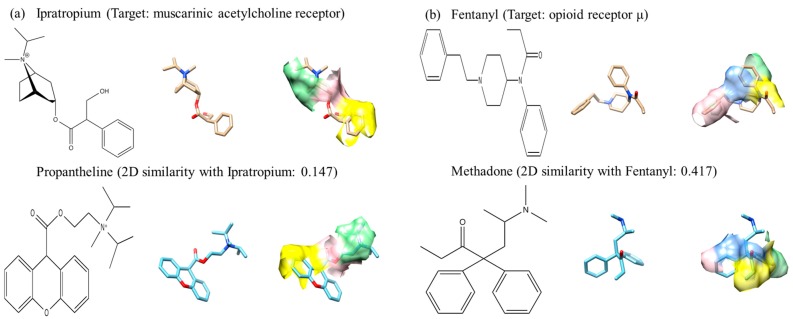
2D and 3D structures of active compounds that PS ranked high relative to other programs. Fifty conformations were generated for each compound. (**a**) The template compound is ipratropium and the query compound is propantheline. PS, USR, GZD, and ROCS ranked the query compound as third, 92nd, 86th, and 122nd, respectively; (**b**) The template compound is fentanyl and query compound is methadone. The rankings from all programs are fifth, 127th, 66th, and 168th for PS, USR, GZD, and ROCS, respectively. Template and query compounds are colored in gold and cyan, respectively. Matched pairs by PS have the same color codes.

To illustrate potential advantages of PS, in Figure 4 we show two examples where PS performs significantly different from the other three methods. In Figure 4, similar surface patch pairs found by PS are shown in the same colors. Figure 4a is an example where PS performed very well in identifying an active compound that is substantially different from a template ligand. The template ligand, ipratropium bromide, and an active ligand found by the search, propantheline, have little two-dimensional structural similarity with a SIMCOMP score of 0.15. PS ranked propantheline as the third most similar compound to the template, while the other programs ranked it lower than 80 among 868 compounds. The program finds patch pairs near nitrogen (colored in green) although their chemical structures are markedly different. The next example, Figure 4b, is a similar case in that a template (fentanyl) and a compound found by the search (methadone) have marginal two-dimensional similarity of 0.42 in the SIMCOMP score. PS ranked methadone fifth whereas the three global shape programs, USR, GZD, and ROCS, ranked it as 127th, 68th, and 168th, respectively. PS recognized similarity around carbonyl oxygen and benzene rings (the yellow and green regions), respectively, in the two molecules. There are cases where PS performed worse than the other three global methods. For example, EF_10%_ generated by PS for the target D-Ala-D-Ala carboxypetidase was 0.9 while the other programs showed values greater than 2.0. These results show that PS’s performance is often complementary to conventional global methods, which suggest that effective combination of the two types of methods may yield substantial improvement to LBVS.

### 4.2. Change of Performance after Removing Similar Compounds

Next, to examine the potential of applying programs in scaffold hopping, active compounds that are similar to the template ligand were removed. The similarity was computed with SIMCOMP [9], which represents compounds as 2D graphs and computes the size of the maximum common subgraph between two compounds as the similarity measure. The similarity threshold values used were 0.75, 0.66, and 0.5. As the similarity threshold value decreases, the number of compounds that are removed increases. The number of maximum conformations generated for a compound was set to 50. The results are summarized in Table 3.

Removing similar compounds makes it harder for a method to find active compounds. As more compounds were removed, both EF and AUC values declined. For all the metrics, ROCS performed the best, although it showed the largest decrease in EF_2%_ when the compounds were removed with a SIMCOMP cutoff of 0.5. EF_2%_ of ROCS decreased from 20.0 to 10.1 (49.5% decrease), USR decreased from 10.0 to 3.5 (65.0% decrease), GZD changed from 13.4 to 6.0 (55.2%), and PS changed from 10.7 to 6.2 (42.1% decrease). In terms of the percentage of decrease, USR performed the worst while PS performed the best.

**Table 3:** Enrichment factors at 10%, 5%, and 2% and AUC values of each method after removing similar active compounds from the dataset.

	EF_2%_	EF_5%_	EF_10%_	AUC
**All Active Compounds**				
USR	10.0	6.2	4.1	0.76
GZD	13.4	8.0	5.3	0.81
PS	10.7	6.6	4.9	0.78
ROCS	20.1	10.7	6.2	0.83
**Similarity < 0.75**				
USR	5.3	4.7	3.4	0.721
GZD	8.5	6.4	4.7	0.775
PS	8.2	5.2	4.2	0.758
ROCS	15.6	9.4	5.6	0.801
**Similarity < 0.66**				
USR	5.9	3.9	3.0	0.652
GZD	7.5	5.6	4.4	0.740
PS	7.9	4.8	3.9	0.736
ROCS	13.6	8.6	5.3	0.764
**Similarity < 0.50**				
USR	3.5	3.0	2.0	0.621
GZD	6.0	4.3	3.5	0.719
PS	6.2	4.2	3.5	0.710
ROCS	10.1	7.9	4.3	0.727

### 4.3. Consensus Methods

In this section, we combined two methods and investigated if the combined methods improve the performance of identifying active compounds. In combining two methods, first the similarity scores of each method were converted into Z-scores. Then, compounds in the library were ranked according to the sum of the two Z-scores from the two combined methods. Results of all six combinations of the four programs were listed in Table 4. Fifty conformations were generated for each compound.

**Table 4:** Enrichment factors (EF) at 10%, 5%, and 2% for combined methods.

Combined Programs	2%	5%	10%
USR + GZD	13.7	7.7	4.7
USR + PS	13.1	7.9	5.0
USR + ROCS	17.1	9.1	5.4
GZD + PS	16.0	9.1	5.9
GZD + ROCS	20.3	10.8	5.3
PS + ROCS	20.5	10.7	6.4

The combination of PS and ROCS, which were the two best methods in Table 1, performed best among the six combinations. This combination has a larger EF than any individual method. All combinations including PS or ROCS show an improved result compared to the results of its partner method. It is observed that combinations including PS are improved from the PS result, while any mixture including ROCS does not make improvements from the ROCS result except for the combination with PS. This is also shown in the statistical test, the student’s *t*-test performed for EF_2%_ shown in Table 5. Improvement by the combination with PS is always statistically significant compared to the PS result, but the improvements from ROCS are not significant for almost all cases.

**Table 5:** Student’s *t*-test between consensus method and individual methods (EF_2%_).

Combined Programs	Single Program	*t*-Value	Single Program	*t*-Value
USR + GZD	USR	1.414	GZD	0.150
USR + PS	USR	1.373	PS	1.369
USR + ROCS	USR	**2.489**	ROCS	1.409
GZD + PS	GZD	1.409	PS	**2.014**
GZD + ROCS	GZD	**2.402**	ROCS	0.137
PS + ROCS	PS	**3.547**	ROCS	0.452

The *t*-values for improvements of the combined methods from each individual method are shown. *t*-values at *p*-value = 0.1 and *p*-value = 0.05 are 1.302 and 1.682, respectively. A *t*-value are shown in bold if it is larger than 1.682 (*i.e.*, statistically significant at *p*-value = 0.05) and underlined if it is larger than 1.302 (*i.e.*, statistically significant at *p*-value = 0.1).

### 4.4. Computational Speed Comparison

Finally, we examined the computational time required for each method (Table 6). Computational time can be a critical issue when the size of a compound library becomes huge. For each method, the time for comparing a template compound against 850 compounds was reported. A single conformation was generated per ligand. The reported computational time does not include time spent for input preparation, *i.e.*, 3DZD computation for GZD and PS and computing the Gaussian volume for ROCS. ROCS needed the longest time because it superimposes two molecules by maximizing volume overlap while USR and GZD take the shortest time because they only compare a one-dimensional vector. It has to be noted here that USR and GZD are coded in Python, slower to execute than binary programs compiled from C++ or Fortran codes, which PS and ROCS adopt.

**Table 6:** Computational time for calculating similarity between 850 compounds and a template compound.

Programs	Time (s)
USR	2.1
GZD	2.3
PS	4.4
ROCS	5.1

## 5. Conclusions

In this article, we have reviewed ligand 3D shape comparison methods and benchmarked representative methods selected from different categories, USR from atomic distance-based methods, GZD from surface-based methods that do not require structure alignment, ROCS from Gaussian function-based methods, and PS from local shape-based methods. 3D methods are aimed at finding compounds with similar shape and surface properties. However, a challenge for 3D methods over 1D and 2D methods is the treatment of conformational flexibility.

From the benchmark results, ROCS performed best in all experiments although its calculation speed is the slowest among all tested programs. USR and GZD are the fastest methods although their performances are lower than ROCS. The Patch-Surfer results showed several distinguishing characteristics. PS did not show a drop in performance as large as the other global methods when the number of maximum ligand conformations was reduced or similar compounds were removed from the compound library. The results of consensus methods in Table 4 showed the potential of combining global and local methods.

As drug discovery turns to less studied targets, new approaches are needed for virtual screening methods that can provide additional benefits compared to conventional methods. Local surface-based methods such as Patch-Surfer may offer advantages, either used alone or in combination with other global methods.
